# Sleep

**Published:** 1883-05

**Authors:** 


### SLEEP.

I THINK the intellectual and moral connections of sleeping have not been sufficiently appreciated. Men and boys have been praised for "burning the midnight oil." Now this "midnight oil" is a delusion and a snare. The student who is fast asleep at eleven o'clock every night and wide awake at seven o'clock every morning is going to surpass another student of the same intellectual ability who goes to bed after twelve and rises before five. In sleep the plate on which the picture is to be taken is receiving its chemical preparation, and it is plain that that which is the best prepared will take the best picture.

Men who are the fastest asleep when they are asleep are the widest awake when they are awake.

Great workers must be great resters.

Every man who has clerks in his employ ought to know what their sleeping habits are. The young man who is up till two, three and four o'clock in the morning, and must put in his appearance at the bank or store at nine or ten o'clock and work all day, cannot repeat this process many days without a certain shakiness coming into his system, which he will endeavor to steady by some delusive stimulus. It is in this way that many a young man begins his course to ruin. He need not necessarily have been in bad company. He has lost his sleep, and losing sleep is losing strength and grace.

Here is the outline of the history of a suicide within my own knowledge. A young man, a stranger in New York, in a good situation, in a large boarding-house, has pleasant young companions; spends his evenings out; goes to midnight parties, from eleven to seven; his nerves become disturbed, then a little drink—a little mistake in business—another drink—reproof from employer—more drink—more mistakes—loss of situation—no help from frivolous companions—money all gone—then credit all gone—then turned out of the boarding-house—wandering in the street—mortification—desperation—shoots himself.

---

HE that has never known adversity is but half acquainted with others or with himself. Constant success shows us but one side of the world, for, as it surrounds us with friends who will tell us only our merits, so it silences those enemies from whom alone we can learn our defects.

